# A proteome-wide screen of *Campylobacter jejuni* using protein microarrays identifies novel and conformational antigens

**DOI:** 10.1371/journal.pone.0210351

**Published:** 2019-01-11

**Authors:** Jiayou Liu, Jodi R. Parrish, Julie Hines, Linda Mansfield, Russell L. Finley

**Affiliations:** 1 Center for Molecular Medicine & Genetics, Wayne State University School of Medicine, Detroit, Michigan, United States of America; 2 Department of Microbiology & Molecular Genetics, Michigan State University, East Lansing, Michigan, United States of America; 3 Department of Microbiology, Immunology, and Biochemistry Wayne State University School of Medicine, Detroit, Michigan, United States of America; New York State Department of Health, UNITED STATES

## Abstract

*Campylobacter jejuni* (*C*. *jejuni*) is a foodborne intestinal pathogen and major cause of gastroenteritis worldwide. *C*. *jejuni* proteins that are immunogenic have been sought for their potential use in the development of biomarkers, diagnostic assays, or subunit vaccines for humans or livestock. To identify new immunogenic *C*. *jejuni* proteins, we used a native protein microarray approach. A protein chip, with over 1400 individually purified GST-tagged *C*. *jejuni* proteins, representing over 86% of the proteome, was constructed to screen for antibody titers present in test sera raised against whole *C*. *jejuni* cells. Dual detection of GST signals was incorporated as a way of normalizing the variation of protein concentrations contributing to the antibody staining intensities. We detected strong signals to 102 *C*. *jejuni* antigens. In addition to antigens recognized by antiserum raised against *C*. *jejuni*, parallel experiments were conducted to identify antigens cross-reactive to antiserum raised against various serotypes of *E*. *coli* or *Salmonella* or to healthy human sera. This led to the identification of 34 antigens specifically recognized by the *C*. *jejuni* antiserum, only four of which were previously known. The chip approach also allowed identification of conformational antigens. We demonstrate in the case of Cj1621 that antigen signals are lost to denaturing conditions commonly used in other approaches to identify immunogens. Antigens identified in this study include those possessing sequence features indicative of cell surface localization, as well as those that do not. Together, our results indicate that the unbiased chip-based screen can help reveal the full repertoire of host antibodies against microbial proteomes.

## Introduction

Bacterial infections continue to claim lives and burden health care systems worldwide. With the rise in antibiotic resistance, the need for new vaccines and diagnostics has become urgent [1, 2]. New vaccines and diagnostic assays can be difficult to develop, in part because bacterial pathogens can encode thousands of proteins, possess complex antigen profiles, and elicit complex immune responses. Although sometimes effective, whole cell vaccines may cause significant side effects. Whole cell vaccines are contraindicated, for example, for *Campylobacter jejuni*, the leading bacterial cause of foodborne gastroenteritis [3] [4]. This is because certain lipo-oligosaccharides on the cell surface of *C*. *jejuni* are thought to induce autoantibodies that can lead to Guillain-Barre Syndrome, a neurological disease that follows *C*. *jejuni* infection in some patients [5, 6]. An alternative to whole cell vaccines is to develop subunit vaccines comprised of a defined subset of microbial antigens that stimulate protective immunity but avoid adverse side effects. A first step to developing such vaccines is to identify specific antigens that illicit an immune response. Well-defined antigen markers would also be useful for serodiagnostic purposes [7, 8].

Various experimental and computational methods have been used to identify or predict *C*. *jejuni* immunogenic antigens and vaccine candidates. Early studies looked for proteins in *C*. *jejuni* whole cell lysates or outer membrane protein fractions that were recognized by antibodies in infected animals or patients [9, 10]. Specific protein antigens were similarly identified and further studied after biochemical purification or after subcloning and expression of the specific genes encoding them [11–17]. Several candidate antigens have been identified and further studied based on their predicted properties such as localization to the outer membrane or secreted fractions [16, 18–23]. Individual candidate immunogens for vaccines have also been identified based on prediction algorithms that screen global protein sequences for localization signals, antigenicity, homology, and other properties [24–28]. While some of the proteins predicted by these algorithms have been identified as immunogens in other screens, most have not been directly tested yet. Finally, antigens have also been identified by screening libraries of *C*. *jejuni* proteins expressed in *E*. *coli* using, for example, animal antisera raised against whole *C*. *jejuni* [7, 29–32].

An alternative approach to identify antigens is to use antisera from patients or infected animals to screen a microarray of individually purified proteins [33–35]. Such an approach has been used to screen random clones of a *C*. *jejuni* expression library, which identified several known and new antigens [36]. A potentially more comprehensive approach is to use proteome-wide microarrays, which are unbiased and do not depend on the natural expression level of the proteins since they are expressed and purified individually. The proteome-wide microarray approach has been used to screen for antigens in the proteomes of yeast [37], *E*. *coli* [38–40], *Bacillus anthracis* [41] and others [42], but not yet for *C*. *jejnui*. In light of the potentially incomplete representation of *C*. *jejuni* antigens in previous studies, we set out to achieve an unbiased screen of the *C*. *jejuni* proteome using a microarray platform.

## Materials and methods

### High throughput expression and purification of proteins

*E*. *coli* BL21 strains that express GST-tagged *C*. *jejuni* fusion were described previously [43]. 5 μL of the stock strains were inoculated into 300 μL of LB medium containing 100 μg/mL carbenicillin and 1% glucose, and were grown overnight at 37°C. 100 μL of each overnight culture was transferred from a well of a 96-well plate to a well of a 24-well 10-mL plates containing 6 mL of the same medium. This resulted in dilution to OD_600_ of ~ 0.2. The diluted cultures were incubated at 28°C until the OD_600_ reached ~0.8. IPTG (Thermo Fisher, Waltham, MA) was then added to a final concentration of 1 mM and the culture was incubated for another 2 hours for most proteins, or 1.5 hours for predicted membrane and secretory proteins. Cells were spun down and frozen.

Cell pellets in the 24-well plates were resuspended and lysed in 160 μL of Novagen BugBuster lysis buffer (Millipore Sigma, Burlington, MA) supplemented with 100 μg/mL lysozyme, 8 μg/mL, DNase 2 mM MgCl_2_, 10 mM NaF, 1 mM Na_3_VO_4_, protease inhibitor tablets (Roche Applied Science, Indianapolis, IN), 500 mM PMSF, 0.1% β-mercaptoethanol, and 10% glycerol. Cells were disrupted by shaking for 30 min at 200 rpm. The lysates were then transferred to Whatman 96-well glass fiber-filter plates (Millipore Sigma) and filtered by spinning. The cleared lysates were transferred to another set of filter plates (sealed at the bottom) that contained 20 μL of glutathione-beads (GE-Healthcare, Chicago, IL) equilibrated in 200 μL of PBS lysis buffer (PBS supplemented with 2 mM EDTA, protease inhibitor tablets, 500 mM PMSF, 0.1% β-mercaptoethanol, 0.2% Triton X-100, and 10% glycerol). The plates were then sealed at the top and rotated for 1 hour at 4°C to allow binding of fusion proteins to the beads. After binding, lysates were spun off the filter plates. The beads were washed by 5 changes of washing buffers: once by PBS lysis buffer, twice by a high salt buffer (PBS supplemented with 2 mM EDTA, 0.2% Triton X-100, 10% glycerol, and 500 mM NaCl) and twice by a low salt buffer (PBS supplemented with 1 mM EDTA, 0.01% Tween-20, 20% glycerol). Proteins were then eluted from the beads with 100 μL of elution buffer (PBS with 1 mM EDTA, 0.01% Tween-20, 40% glycerol) supplemented with 40 mM reduced glutathione. Finally, excessive glutathione was removed, and proteins were concentrated about 3-fold by centrifugation of the protein preps in 96-well Ultracel-10 filter plates (MilliporeSigma) with a 10 kDa size cut-off, and re-centrifugation after adding an elution buffer that contained no glutathione. High throughput execution of the above procedure was facilitated by a Biomek FX liquid handling robot (Beckman Coulter Life Sciences, Indianapolis, IN).

### Protein microarray construction and antibody staining

Polyacrylamide hydrogel slides (PerkinElmer, Waltham, MA) were hydrated for 30 min in 3 changes of distilled water and dried in a 42°C oven for 1 hour. Protein samples were printed in duplicate onto the hydrogel using a contact arrayer under the conditions of 80% humidity and low temperature (15°C). The proteins were then allowed to immobilize onto the hydrogel matrix by leaving the slide in a humidified dish overnight at 4°C. The hydrogel had previously been shown to be superior among many other coatings tested in terms of low background and high signal to noise ratio [44]. The protein microarray was washed briefly in PBST buffer (PBS with 0.1% Tween-20), blocked in 1% BSA for 1 hour, and again washed briefly. An aliquot of antiserum (rabbit or mouse at 200-fold dilution) was applied onto the array together with 1% BSA and incubated for 2 hours in a humidified dish at 4°C. After incubation, the array was washed three times for 20 min each in PBST. To probe for rabbit antibody binding, the microarray was then incubated, under the same condition as for primary antibodies, with a detection mixture that contained 1 μg/mL of Cy5-labeled goat anti-rabbit IgG (see labeling protocol below), as well as 30 μg/mL of fluorescein-labeled goat anti-GST antibody (Rockland, Limerick, PA) for dual detection of GST-fusion proteins. To probe for mouse antibody binding, anti-mouse IgG biotin conjugate (Millipore Sigma) was used at 200-fold dilution, followed by Cy5-streptavidin conjugate (GE Healthcare) at 200-fold dilution, together with fluorescein-labeled goat anti-GST antibody as before. After incubation, the arrays were washed extensively before scanning. Rabbit antisera raised against whole cells (not heat- or formalin-killed) of *C*. *jejuni* ATCC 29428, against various O and H strains of *Salmonella*, against various O and K antigenic serotypes of *E*. *coli* were all purchased from Fitzgerald (Concord, MA). Mouse antiserum was from mice orally challenged with *C*. *jejuni* strain NCTC11168 (24 days post infection) as described [45]. Pooled human sera from 10 healthy individuals (cat. no. IPLA-SER) was obtained from Innovative Research (Novi, MI). Purified GST [46] was used as standard.

### Protein labeling

Each labeling reaction contained 10 μL (~ 5μg) of goat anti-rabbit IgG (Promega, Madison, WI), 50 μL of carbonate buffer at pH 9.0, and 25 μg of N-hydroxysuccinimide ester-linked Cy5 (GE Healthcare). After the reaction had proceeded for 2 h at room temperature in the dark, 50 μL of 1 M Tris-HCl (pH 8.5) were added and the incubation was continued for another 30 min to quench the remaining free Cy5. The quenched Cy5 was then removed using a size-exclusion chromatography Bio-Spin P6 spin column (Bio-Rad, Hercules, CA) with a size cut-off of 6,000 Da.

### Microarray data analysis

Slides were scanned using a laser scanner (Genetix aQuire,) at 10 μm resolution. The various antisera were detected in the Cy5 channel while the fluorescein-labeled anti-GST was detected in the Cy3 channel. Fluorescence intensities were collected using the aQuire software after manually optimizing spot registration. Empty spots were excluded; for the remaining spots, correlation of median intensity values vs. mean intensities was examined using R^2^. If R^2^ was below 0.9, the scanned image was re-examined to adjust ring registration. All raw data is available at ArrayExpress (www.ebi.ac.uk/arrayexpress/), accession number E-MTAB-7535. The data sets were treated as follows: For each spot the fold increase in antisera fluorescence (Cy5) intensity over GST protein fluorescence (Cy3) intensity (FIp) was calculated as the ratio of mean background-subtracted Cy5 intensity per pixel over the mean background-subtracted Cy3 intensity per pixel, normalized (divided) by the ratio of total Cy5/Cy3 intensities per slide. In addition, the signal to noise ratio of Cy5 fluorescence for each spot was also calculated, here referred to as the fold-increase of antigen signal over background, or FIb. The average FIp or FIb of replicates for each protein was calculated. Proteins with average FIp and FIb more than 3 standard deviations above the negative control proteins (pVIR proteins and GST only) were considered positive. FIp was also used to compare antigen reactivities to different antisera. Antigens for which FIp was greater than 2 for *E*. *coli* or *Salmonella* antiserum were considered cross-reactive.

### Preparation of protein fractions

*C*. *jejuni* strain ATCC 29428 was obtained from the American Type Culture Collection, and grown in Brucella broth or on Mueller Hinton agar (both from BD, Franklin Lakes, NJ) under microaerophilic conditions (2.5% O_2_, 7.0% H_2_, 10% CO_2_, balance of N_2_) in a sealed plastic bag at 37°C. Bacteria were harvested from 2-day old broth culture by microcentrifugation at 7,000 rpm for 10 min and washed in 0.15 M NaCl before another centrifugation. Supernatants were collected from these two centrifugations and cleared by spinning at 11,000 rpm for 10 min. The cleared supernatant, enriched for secretory proteins, was dialyzed against distilled water and concentrated in a speed-vac. The saline-washed bacteria were incubated in 0.2 M glycine-HCl, pH 2.2, for 15 min at room temperature before spinning at 11,000 rpm for 10 min. The supernatant, representing acidic glycine-extractable surface proteins, was neutralized, dialyzed, and concentrated as above. To prepare the membrane protein fraction, bacteria were harvested from agar plates and washed 3 times in PBS before being suspended in PBS lysis buffer (see above). The bacteria were then sonicated, and the resulting lysate was cleared by centrifugation twice at 10,000 x g for 20 min. The cleared lysate was then subject to ultracentrifugation at 100,000 x g for 2 hours. The pellet, considered enriched for membrane proteins, was suspended in distilled water; the supernatant was used as the membrane-depleted fraction enriched for intracellular proteins.

### Immunoblotting and antibodies

Standard protocols for SDS-PAGE and immunoblotting were used. To run non-denaturing PAGE, SDS was omitted from the gel and from the sample buffer and the proteins were also spared from heat treatment prior to electrophoresis. To make antibodies recognizing the denatured form of Cj1621, GST-fusion protein for Cj1621 was fully denatured by heating twice in SDS sample buffer at 100°C for 10 min. After electrophoresis, gel pieces containing the full-length fusion protein bands visualized after incubation with saturated KCl were cut out and used to immunize rabbits by Open Biosystems (Huntsville, AL). The resulting antiserum possessed a high titer specific to Cj1621 and was used to detect Cj1621 on immunoblotts without purification.

## Results

### Protein microarray construction

We previously cloned over 90% of the *C*. *jejuni* open reading frames (ORFs) into a vector for expression of N-terminally-tagged GST fusion proteins in *E*. *coli* [43]. To construct protein microarrays we individually expressed and purified 1406 of the GST fusion proteins, representing 86% of the ORFs, in addition to 54 clones expressing ORFs encoded by the *C*. *jejuni* pVIR plasmid [47]. Proteins were purified under native conditions using a high throughput protocol optimized to reduce degradation of membrane proteins and formation of inclusion bodies (see Methods). Protein purity was confirmed by Coomassie staining for randomly selected samples (S1 Fig). Proteome arrays were constructed by printing all protein samples in duplicate onto single slides coated with hydrogel.

**Fig 1:**
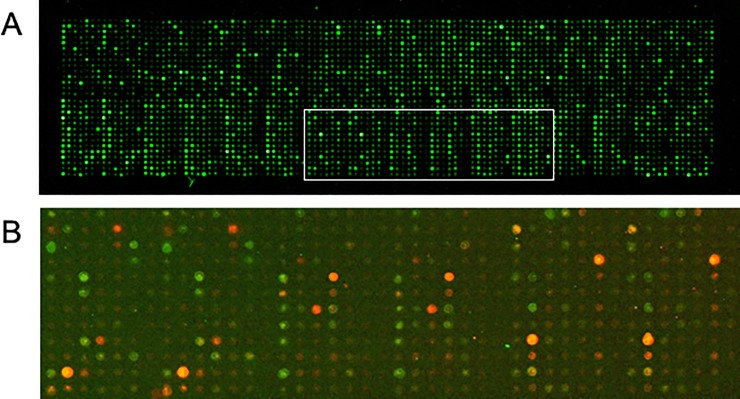
Microarrays. **(A)** Protein microarray with 1460 purified GST-fused *C*. *jejuni* proteins duplicated on a slide with 3,136 spots, including spots with purified GST and blank controls. Duplicate groupings are every 7 rows (oriented vertically). The microarray was probed with fluorescein-labeled anti-GST antibodies (green). (**B)** A section of the protein microarray (corresponding to the boxed region in Fig 1A) probed with fluorescein-labeled anti-GST antibodies (green) and rabbit anti-*C*. *jejuni* antisera plus Cy5-labeled goat anti-rabbit IgG (red).

**Fig 2:**
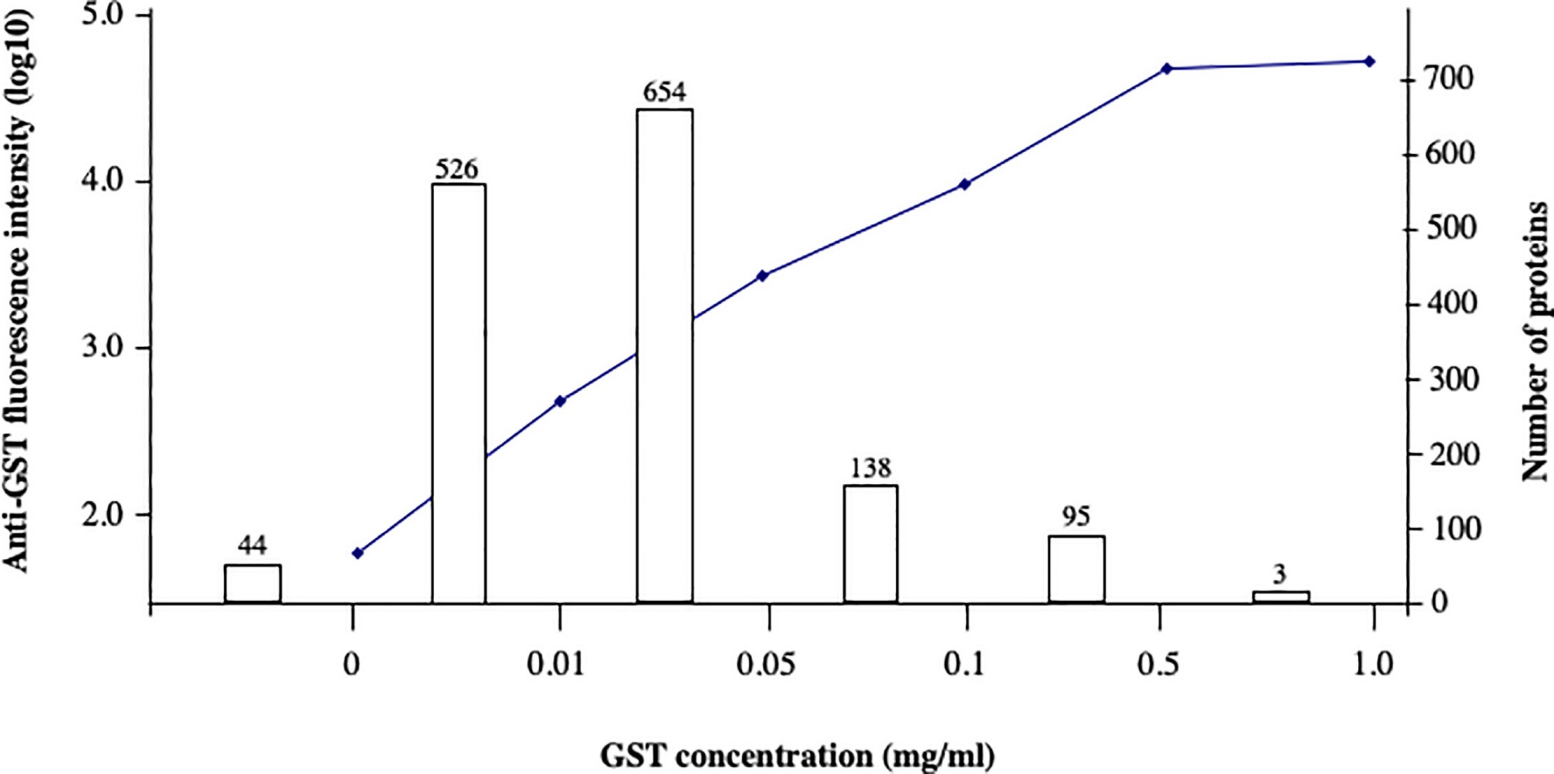
Arrayed protein concentrations. The curve depicts anti-GST fluorescence intensities (log10, left Y axis) for GST standards at the indicated concentrations (X axis); “0” GST corresponds to spots with no added protein (“Empty” in Fig 3). Bars depict the number of *C*. *jejuni* proteins with anti-GST fluorescence intensities corresponding to each range of GST concentrations.

To quantify proteins printed on the array, serial dilutions of a GST standard were also printed and the array was probed using an anti-GST antibody (Fig 1A). The anti-GST fluorescence intensities for the standard series appeared linear in the range of 0.01 to 0.5 mg/mL, with the intensity value for 0.01 mg/mL of GST, the lowest dilution, significantly above that for background signal of spots with no GST (Fig 2). The intensity value for GST at 1 mg/mL was lower than estimated from linear extrapolation, due to saturated staining. 97% the purified *C*. *jejuni* proteins had GST signals above the background signal (corresponding to 0 mg/mL of GST), while 61% were above the signal for 0.01 mg/mL. Protein spots at 0.01 mg/mL would correspond to a few picograms per sample typically printed on the slide.

### A screen for immunogenic antigens on the microarray

We screened the microarray for proteins recognized by antiserum from rabbits exposed to *C*. *jejuni*. The commercial antiserum (Methods) was raised against whole cells of *C*. *jejuni* strain ATCC 29428 using a proprietary formulation that did not kill the cells by heat or formalin but potentially maintained proteins in their native states. This antiserum reproducibly recognized a subset of the *C*. *jejuni* proteins on the microarray with a wide range of intensities, while it did not recognize various concentrations of BSA and GST spotted on the array (Fig 1B and below). Protein preparations representing the 54 pVIR-encoded proteins did not stain above background levels with the antisera. This is consistent with the observation that pVIR appears absent from the ATCC 29428 strain, as confirmed by our inability to detect pVIR genes from this strain by PCR (data not shown).

To measure reproducibility, the duplicated sets of intensity values for all data points were plotted (S2 Fig). A correlation value of 0.96 was obtained for duplicates within an array. High reproducibility was also observed for GST staining for replicates both within and between arrays in parallel experiments. Reproducible dual staining of GST fusions suggests that, by normalizing to protein amounts measured by GST staining, antigen signals can be quantitatively compared across arrays or across parallel experiments.

**Fig 3:**
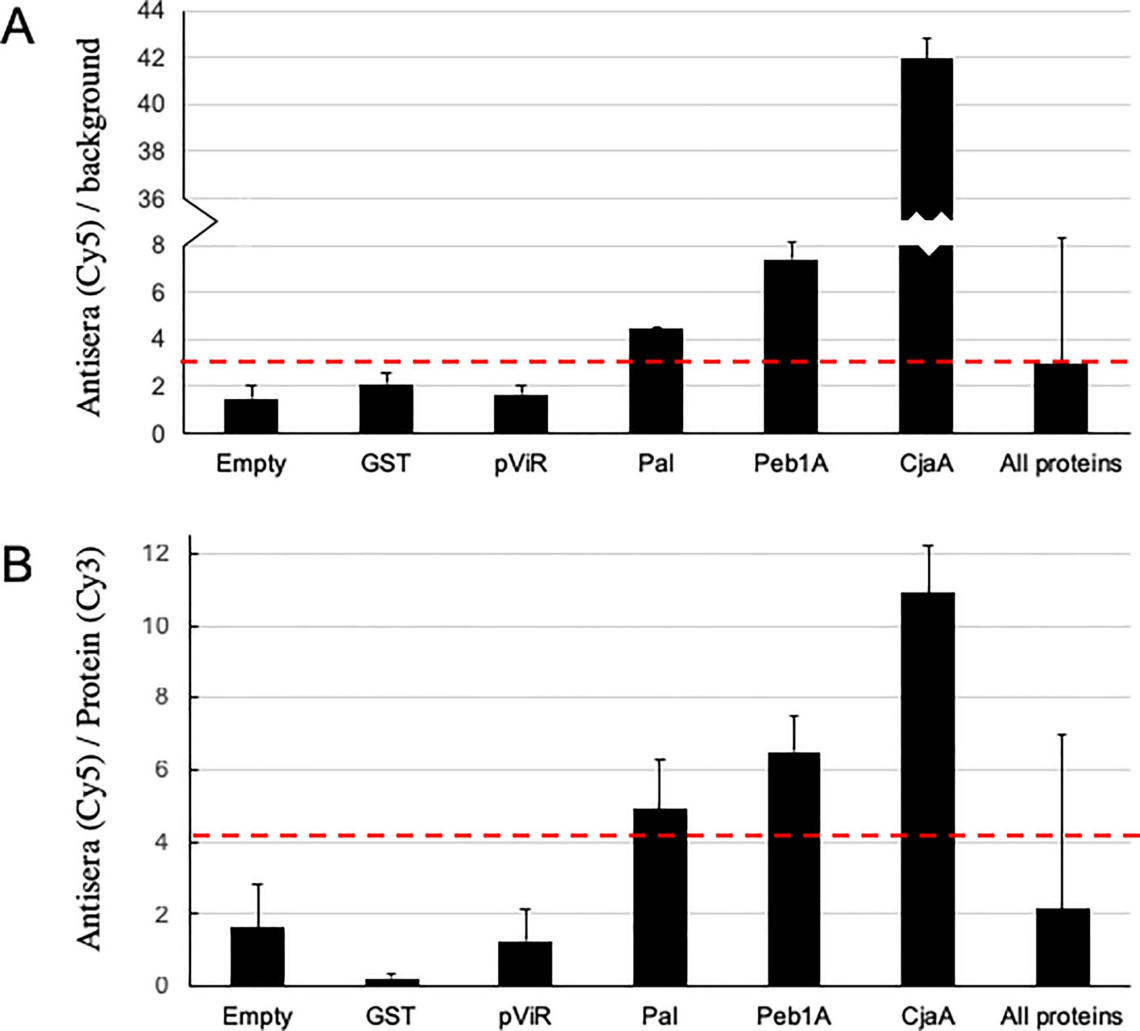
Antigen signals on the microarray. **(A**) The mean ratio (Fib) of anti-campylobacter antisera staining intensities (Cy5) over background staining for spots with the indicated proteins or blanks. Data are from duplicates of: 60 spots with no added protein (Empty); 3 spots with purified GST (GST); 54 spots with the 54 pVIR proteins (pVIR); spots with Pal, Peb1A, or CjaA; and all spots with the remaining 1413 *C*. *jejuni* proteins (All proteins). Error bars are standard deviation. The dotted red line indicates the Fib cut off (>3) for antigens. **(B)** The mean ratio (Fip) of anti-campylobacter antisera staining intensities (Cy5) over GST protein staining (Cy3) for spots with the indicated proteins or blanks as in Fig 3A. Error bars are standard deviation. The dotted red line indicates the Fip cut off (>4) for antigens.

We defined antigens recognized by the anti-*C*. *jejuni* antiserum as those with fluorescence signals that were at least three standard deviations above that for the negative control proteins (GST and pVIR proteins) on the array (Fig 3). This resulted in 102 proteins that exhibited mean anti-*C*. *jejuni* signals over four times that of the corresponding GST signals (FIp) and over three times above the background signal (FIb) (S1 Table). Among these are 13 previously identified antigens, including the well-known immunogenic proteins Pal (peptidoglycan associated protein; also known as CjaD or Omp18) [7, 15, 48, 49], CjaA (*C*. *jejuni* antigen A) [30, 49, 50], and Peb1A [12, 22] (Fig 3). To further define antigens that are immunogenic we probed the microarray with antiserum from a mouse infected with *C*. *jejuni* (Methods) [45]. The mouse and rabbit antisera were raised against *C*. *jejuni* strains NCTC11168 and ATCC29428, respectively, and any immunogenic proteins recognized by the two sera are likely shared between the species. 72 proteins were recognized by the mouse antisera, including 30 of the 102 that were positive with the rabbit anti-*C*.*jejuni* sera (S1 Table). The 30 proteins that were immunogenic in both rabbit and mouse included again the well characterized antigens CjaA, Pal, and Peb1A, as well as previously identified antigens Cj0404 and RplL [31], Tuf [32, 51], and Cbf2/Peb4A[12].

**Fig 4:**
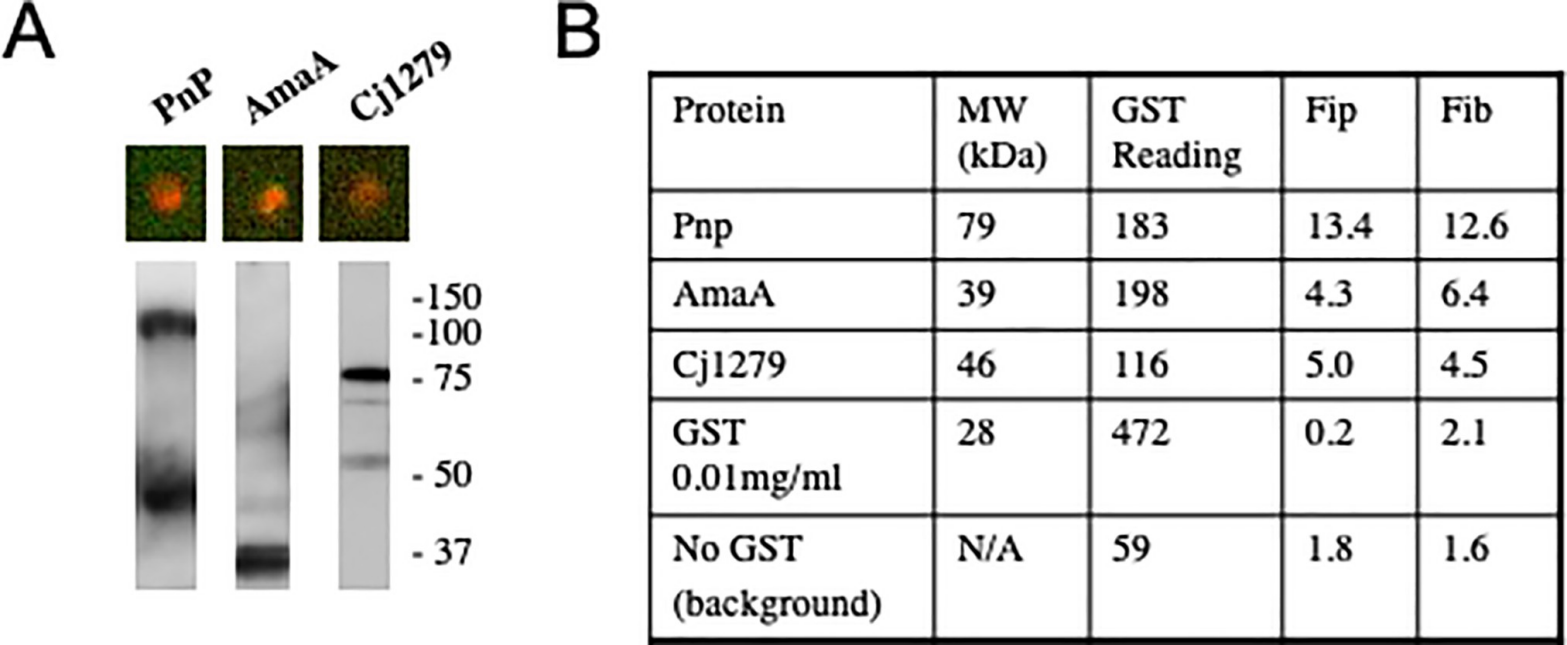
Identification of antigens at low concentrations. (**A)** The indicated proteins were recognized by the rabbit antiserum against *C*. *jejuni* on the array (top) and by immunoblotting (bottom). The array shows dual color probing with anti-*C*. *jejuni* (red) and anti-GST (green). amaA has 7 transmembrane spanning domains and results in a smear at the expected size plus a single band degradation product on the denaturing gel (lane 2). (**B)** The low GST reading on the array (mean background corrected fluorescence intensity/pixel) is compared with that for a known concentration (0.01mg/ml) of GST and a series of 60 spots with no protein (no GST). The microarray values for fold increase of anti-*C*.*jejuni* antisera over anti-GST intensities (Fip) and fold increase of anti-C.jejuni antisera over background intensities (Fib) are also shown. The molecular weight of each protein without the GST fusion is shown.

As shown in Fig 2, close to 40% of the protein samples appeared to have concentrations below 0.01 mg/mL. Our screen identified several antigens from this list of low concentration proteins, including the previously identified antigen, AmaA [29], and others (Fig 4). These data suggest that the screen sensitivity can be determined by the specific antibody titers in the test serum, which may be much more sensitive than the anti-GST antibodies. Thus, while low protein concentrations could lead to missed antigens, given a high titer antiserum, a trace amount of antigen (lower picograms) on the array can enable positive identification.

### Specific antigens

Gastroenteritis can be caused by a number of other bacterial pathogens in addition to *C*. *jejuni*. Strains of *E*. *coli* and *Salmonella*, for example, are among the prominent causes in this part of world. To maximize the potential value of new *C*. *jejuni* antigens for diagnostics or vaccines, we sought to identify antigens that were not cross reactive with antisera against *E*. *coli* or *Salmonella*. We probed the microarrays with rabbit antisera against various O and H strains of *Salmonella* or against various O and K serotypes of *E*. *coli* in parallel with the *C*. *jejuni* antiserum. We found that 68 out the 102 *C*. *jejuni* antigens cross-reacted at least weakly (FIp>2) with the anti-*E*. *coli* or anti-*Salmonella* antisera (S1 Table). This included 9 of the previously identified antigens, including Pal and Peb1A, which both interacted with the anti-*E*.*coli* antisera, as well as FlaB [11] and Tuf [32, 51], which both interacted with all three rabbit antisera. This analysis also identified 34 antigens that were specifically recognized by only the anti-*C*. *jejuni* antisera including CjaA and three other previously identified antigens, AhpC and Adk [52], and AmaA [29] (Table 1). None of the 34 specific antigens reacted to pooled human sera obtained from 10 healthy individuals. Six of the specific antigens were also recognized by antisera from the mouse model of *C*. *jejuni* infection [45], including CjaA and five novel antigens: Cj0144, Cj0262c, Cj1621, GreA, and PrfA (Table 1). Three of these new antigens are predicted to have transmembrane domains (Cj0144, Cj0262c, and Cj1621) and none of them were among the proteins previously predicted to be immunogenic or vaccine candidates based on sequence features [25, 28].

**Table 1:** Specific *C*. *jejuni* antigens identified on the microarray.

Gene Name	Fip^a^	Fib^b^	Rabbit^c^ *C*.*jejuni*	Mouse^c^ *C*.*jejuni*	Rabbit^c^ *E*.*coli*	Rabbit^c^ S.species	Human^c^ Control	Previously identified [ref]
Cj1621	11.8	67.8	1	1	0	0	0	
cjaA	10.9	43.5	1	1	0	0	0	yes [17, 30, 50]
prfA	8.9	13.7	1	1	0	0	0	
Cj0144	6.5	31.8	1	1	0	0	0	
greA	4.8	21.4	1	1	0	0	0	
Cj0262c	4.5	30.8	1	1	0	0	0	
frr	11.6	53.2	1	0	0	0	0	
Cj0355c	11.3	61.5	1	0	0	0	0	
Cj0036	11.2	3.3	1	0	0	0	0	
ychF	9.7	63.7	1	0	0	0	0	
ahpC	9.1	35.5	1	0	0	0	0	yes[52]
pgi	8.9	4.5	1	0	0	0	0	
NT01CJ0080	7.6	3.7	1	0	0	0	0	
clpB	7.6	14.9	1	0	0	0	0	
Cj0159c	7.5	5.2	1	0	0	0	0	
icd	6.8	27.1	1	0	0	0	0	
Cj0771c	6.8	4.9	1	0	0	0	0	
pyk	6.7	10.9	1	0	0	0	0	
fliL	6.6	14.9	1	0	0	0	0	
Cj0092	6.3	7.7	1	0	0	0	0	
selB	6.3	5.5	1	0	0	0	0	
Cj0327	5.7	3.4	1	0	0	0	0	
gyrB	5.5	3.9	1	0	0	0	0	
Cj0152c	5.5	4.2	1	0	0	0	0	
Cj1613c	5.5	48.4	1	0	0	0	0	
petC	5.4	6.2	1	0	0	0	0	
Cj1275c	5.3	3.2	1	0	0	0	0	
adk	5.3	20.6	1	0	0	0	0	yes [52]
Cj0888c	5.2	3.5	1	0	0	0	0	
Cj1279c	5.0	4.5	1	0	0	0	0	
Cj0406c	4.5	4.1	1	0	0	0	0	
Cj0539	4.5	3.4	1	0	0	0	0	
amaA	4.3	6.4	1	0	0	0	0	yes [29]
livK	4.0	3.4	1	0	0	0	0	
Additional positives								
peb4\cbf2	16.4	11.3	1	1	1	0	0	yes [12]
Cj0404	12.1	17.7	1	1	1	1	0	yes [31]
peb1A	6.5	7.3	1	1	1	0	0	yes [12, 22, 50]
rplL	6.3	10.9	1	1	1	1	1	yes [31]
tuf	5.2	12.4	1	1	1	1	0	yes [32, 51]
pal	4.9	4.5	1	1	1	0	0	yes [7, 15, 48–50]

List of antigens specifically recognized by rabbit anti-*C*. *jejuni* antisera but not by other rabbit or human control sera, plus six at the bottom that were not specific but were identified with both the rabbit and mouse anti-*C*. *jejuni* and previously in other studies.

^a^ FIp—fold increase in rabbit anti-*C*. *jejuni* antisera fluorescence (Cy5) intensity over GST protein fluorescence (Cy3) intensity (Methods).

^b^ FIb—fold-increase of rabbit anti-*C*. *jejuni* antisera fluorescence over background (Methods).

^c^ Each of the columns (rabbit anti-*C*. *jejuni*; mouse anti-*C*. *jejuni*, rabbit anti-*E*. *coli*; rabbit anti-*Salmonella*, human control serum) indicates whether the protein was recognized (1) or not (0) by that antisera on the microarray above the cut off (Methods).

### Cj1621 is a novel Campylobacter-specific conformational antigen

**Fig 5:**
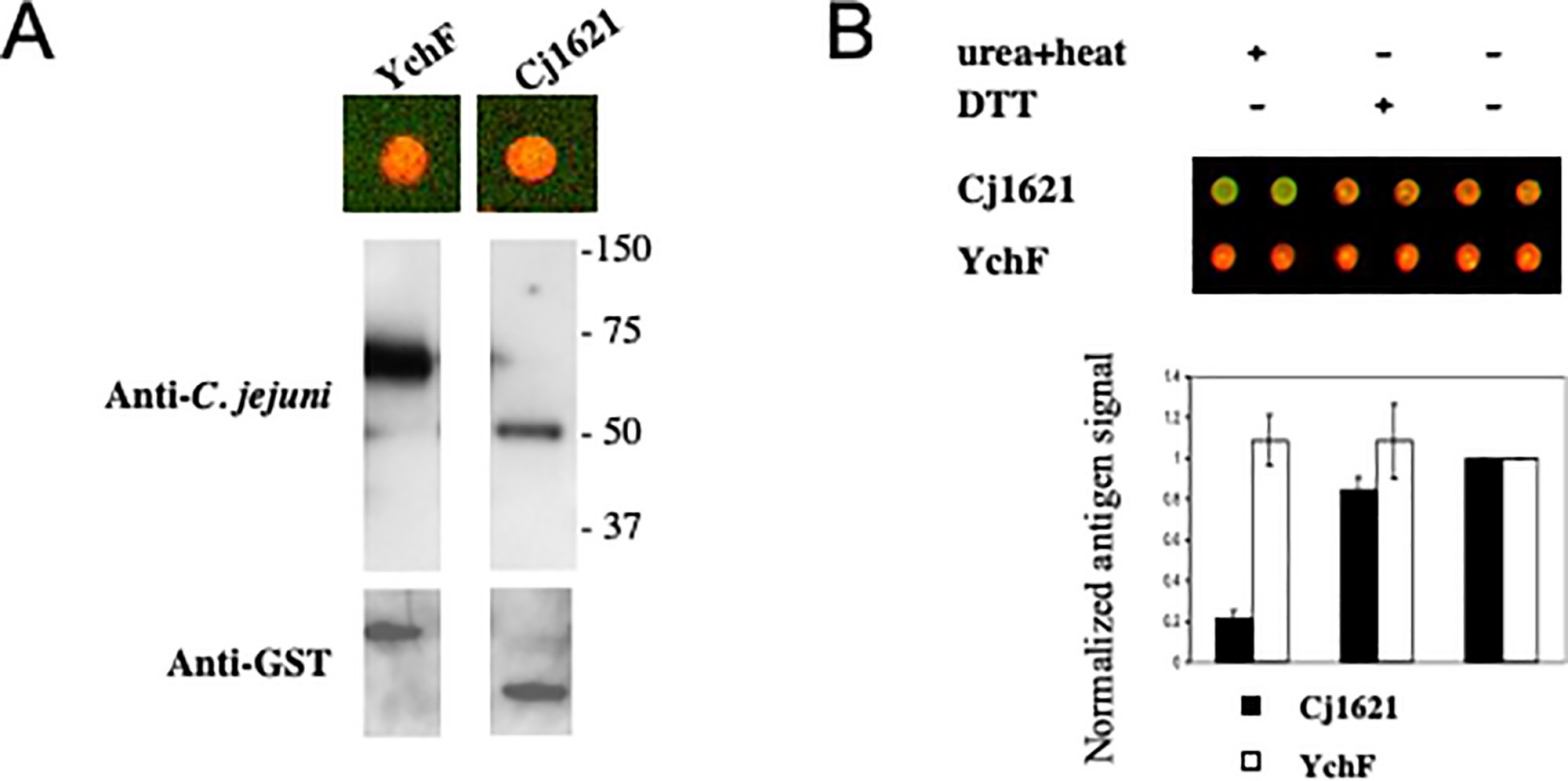
Cj1621 is a conformational antigen. **(A)** Cj1621 and YchF on the microarray (top) have equivalent amounts of proteins by anti-GST staining (green) and are strongly recognized by the rabbit anti-*C*. *jejuni* antisera (red). Immunoblotting on a denaturing gel (bottom) shows that the antisera strongly recognizes YchF but only weakly recognizes denatured Cj1621; the same blot probed with anti-GST shows that similar amounts of the two proteins were loaded. (**B)** The antisera signal (red) of proteins on the array is eliminated by denaturing for Cj1621 but not for YchF; the lower panel is a quantitative representation of the scan image where background-subtracted mean intensity of Cy5 was first normalized to Cy3, then to untreated control. The array images in both (A) and (B) were captured after dual detection (see Methods) of antisera signal (red, Cy5) and GST signal (green, Cy3).

We further analyzed Cj1621 because it produced the strongest antisera signal among the five novel antigens that we identified that were recognized by both the rabbit and mouse anti-*C*. *jejuni* antisera and not by anti-*E*. *coli* or anti-*Salmonella*. To verify that the purified protein is recognized by the antisera, we ran Cj1621 and an unrelated specific novel antigen, YchF, on a denaturing gel and probed with the rabbit anti-*C*. *jejuni* antisera (Fig 5A). The antisera recognized protein bands of the expected sizes for both GST fusions. However, comparing the immunoblots with array data, we noticed a disagreement of signal intensities; whereas YchF and Cj1621 on the microarray were equally well-recognized by the antisera, on the immunoblot the signal for Cj1621 was much weaker (Fig 5A). The weaker antigen signal on the immunoblot could be due to disruption of the epitope either by the reducing agent or by the denaturing conditions. Proteins in the array platform were not subjected to reducing agent or denaturation. To test this hypothesis, we purified the Cj1621 fusion and tested it on the array platform with or without treatment by DTT or by heating in the presence of urea. We found that while the antisera recognized YchF both before and after denaturation, it did not recognize denatured Cj1621. Additional evidence in support of this conclusion was obtained in a parallel experiment using denaturing and nondenaturing PAGE gels with or without SDS treatment of the samples (data not shown). Thus, it appears that Cj1621 contains a so-called conformational epitope that is only recognized in the native, nondenatured protein.

**Fig 6:**
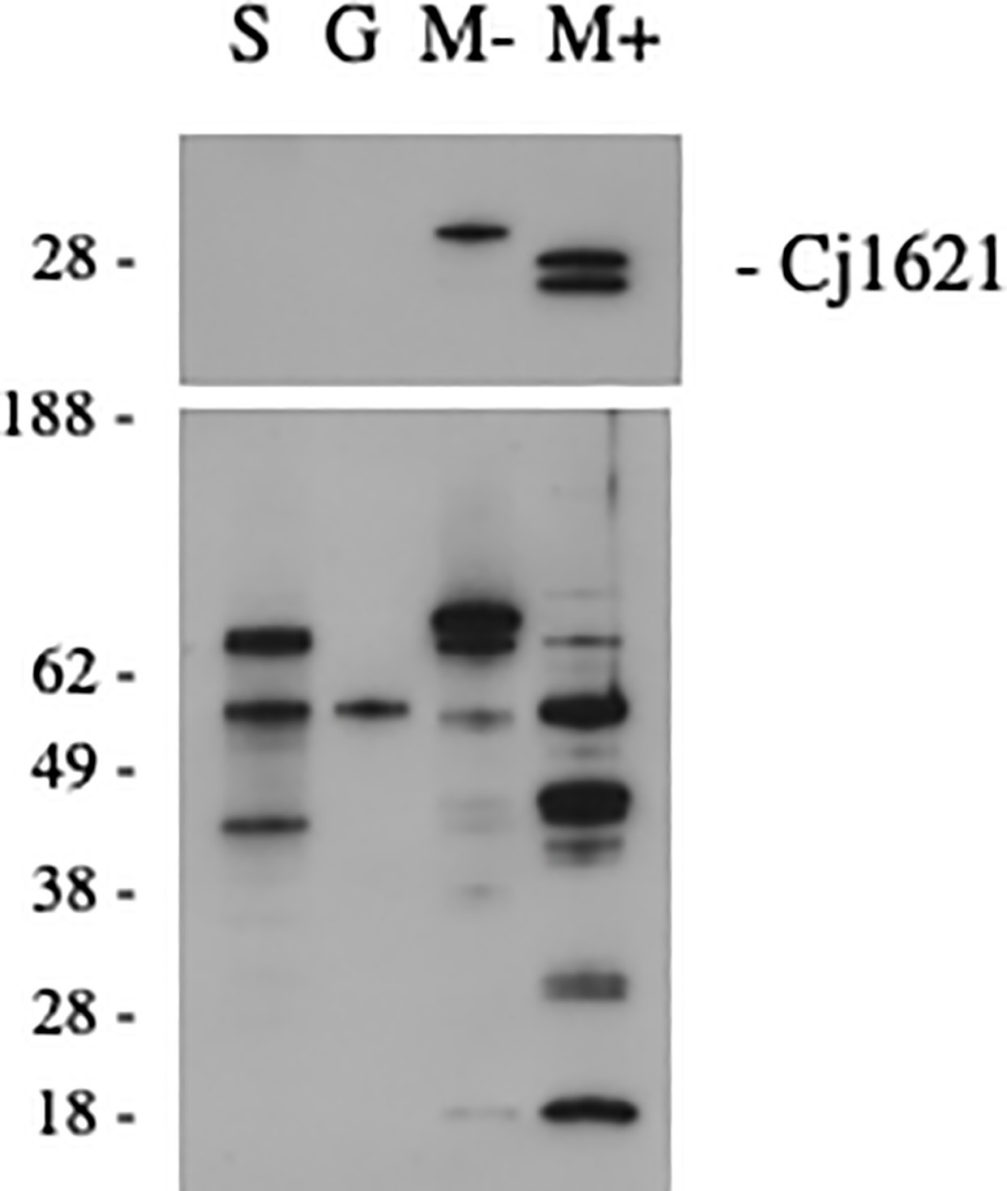
Membrane localization of endogenous Cj1621. Protein fractions were probed with rabbit antiserum raised against denatured recombinant Cj1621 (upper panel) or antiserum from *C*. *jejuni*-infected mouse (lower panel). S. secretory, G. acid glycine extract, M-, membrane depleted or intracellular, M+, membrane enriched.

Cj1621 is a protein of unknown function that appears to have close orthologs only in Campylobacter species. The protein contains a short hydrophobic region at the N-terminus which is usually annotated as a probable signal sequence or as a transmembrane domain [53]. To clarify the localization of Cj1621, we grew *C*. *jejuni* strain ATCC 29428 and prepared various fractions of proteins that were enriched for secretory proteins, cell surface proteins extractable by acidic glycine, membrane proteins, or intracellular proteins. We probed these protein fractions on Western blot using an antiserum raised against the denatured form of Cj1621 (Methods). This antiserum detected a doublet corresponding to endogenous Cj1621 only in the membrane fraction, and more weakly a larger protein in the soluble fraction (Fig 6). Interestingly, while the mouse anti-*C*.*jejuni* antiserum recognizes proteins in all fractions, it recognized the doublet corresponding to Cj1621 only in the membrane fraction. These results support the prediction that Cj1621 is associated with the membrane.

## Discussion

We developed protein microarrays for the purpose of identifying *C*. *jejuni* proteins that are immunogenic in animals. By probing these microarrays with antisera raised against intact *C*. *jejuni* or other microbes, or with antisera from a mouse model of *C*. *jejuni* infection, we identified a list of strongly immunogenic antigens. The approach was validated by the finding that we identified the most highly studied and cited immunogenic *C*. *jejuni* proteins, CjaA, CjaD, Pal, and Peb1A, along with several other known but less well-studied antigens (S1 Table). We also identified many novel antigens that had not been discovered or predicted previously. Our ability to identify new antigens may be attributable to some of the features of the approach. First, it is likely that we screened a more comprehensive set of *C*. *jejuni* proteins than previous studies. One study [36], for example, used a similar microarray approach using proteins purified from randomly selected *E*. *coli* clones, yet the total number of unique proteins screened unclear. In contrast, by individually purifying and arraying proteins we were able to screen a defined and large set of proteins, approaching 86% of all *C*. *jejuni* proteins. Second, the screen was highly sensitive. Even though 40% of the proteins were predicted to be below picogram levels on the microarray, some of them could still be detected by high titer specific antibodies in the test sera. By normalizing signals both to background and to the amount of protein in each spot using anti-GST staining, we were able to identify and quantify reproducible antigen signals over a broad range of concentrations and intensities. Third, we demonstrated that the approach could detect conformational antigens that would not be detected by many of the previous approaches that have been applied to identify antigens. It is expected that immunization or infection of animals or humans with intact living *C*. *jejuni* would result in production of some antibodies that would only recognize intact native proteins. Antigens with such conformational epitopes could only be identified by probing native proteins with antisera raised against intact *C*. *jejuni*. This appears to be the case with Cj1621, one of the strongest specific novel antigens that we identified.

Antigens that are specific for an organism can be useful for development of diagnostics or specific vaccines. Previous methods have addressed specificity at the screen stage by absorption of test serum to unwanted strains in an effort to make the serum more specific (e.g., [32, 51]). Conceivably, however, nonspecific high titer antibodies may be difficult to deplete while some specific low titer antibodies may be lost by preabsorption. We took the simpler approach of probing the microarrays with control antisera from animals inoculated with *E*. *coli* or *Salmonella* species. This identified a large number of the original antigens as cross reactive with either the anti-*E*. *coli* or anti-*Salmonella* sera. Of the antigens recognized by both rabbit and mouse anti-*C*. *jejuni* antisera, 80% (24/30) were cross-reactive with anti-*E*. *coli* or anti-*Salmonella* sera, including some well-known antigens like Pal and Peb1A. These results suggest that although some antigens may be immunodominant, they may not be useful for diagnostic assays or specific vaccine candidates because they are cross reactive. In contrast, our analysis also identified some antigens that may be very useful because they are recognized by only anti-*C*. *jejuni* antisera.

## Conclusions

We used an unbiased microarray approach to identify immunogenic *C*. *jejuni* proteins that may be useful for developing diagnostic assays and vaccines. By probing the microarrays with several different antisera, we were able to identify a subset of antigens that were specifically recognized by rabbit or mouse inoculated with *C*. *jejuni*, but not by other antisera. These included the well-known antigen CjaD and five novel antigens: Cj0144, Cj0262c, Cj1621, GreA, and PrfA. One of these, Cj1621, which appears to contain a conformational epitope, is a Campylobacter-specific protein that is associated with the cell membrane, making it a strong candidate for further study.

## Supporting information

S1 Fig Coomassie staining of 13 randomly-selected protein samples from the collection of purified GST fusion proteins run on SDS-PAGE. Lane 1 is the protein molecular weight marker.
(TIFF)

S2 Fig Scatter plot of duplicated data sets. **A.** Antigen intensities for duplicates within an array. **B.** GST intensities for duplicates between arrays. The antigen and GST signals were obtained in dual detection. Background subtracted mean intensities were used.
(TIFF)

S3 Fig Uncropped images of immunoblots from Figs 4A, 5A and 6. (TIFF)

S1 Table List of proteins on the microarray and the results with various antisera. (XLSX)
