# Supplementary figures and images for: A proteome-wide screen of Campylobacter jejuni using protein microarrays identifies novel and conformational antigens
Source: PLoS One. 2019 Jan 11;14(1):e0210351. doi: 10.1371/journal.pone.0210351 (PMC6329530; doi:10.1371/journal.pone.0210351)

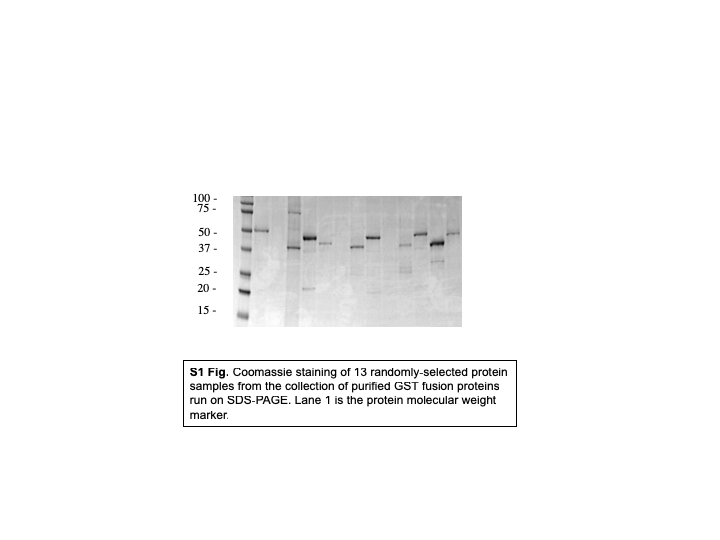

Supplement: S1 Fig — Lane 1 is the protein molecular weight marker. (TIFF) [file pone.0210351.s001.tiff]

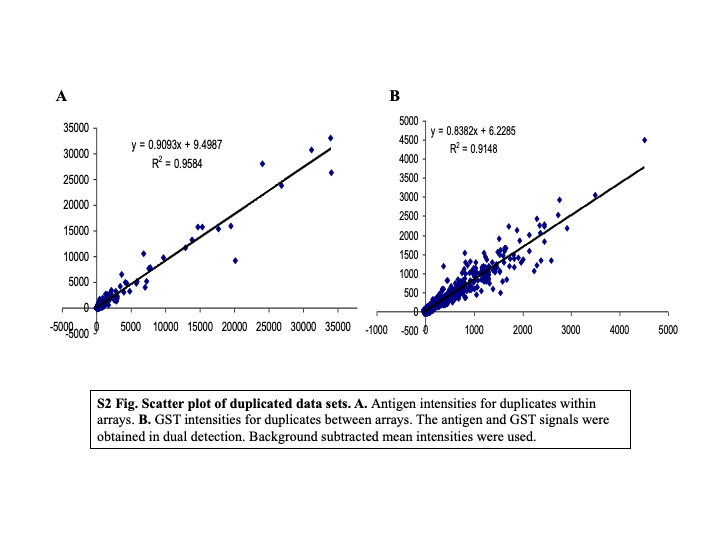

Supplement: S2 Fig — A. Antigen intensities for duplicates within an array. B. GST intensities for duplicates between arrays. The antigen and GST signals were obtained in dual detection. Background subtracted mean intensities were used. (TIFF) [file pone.0210351.s002.tiff]

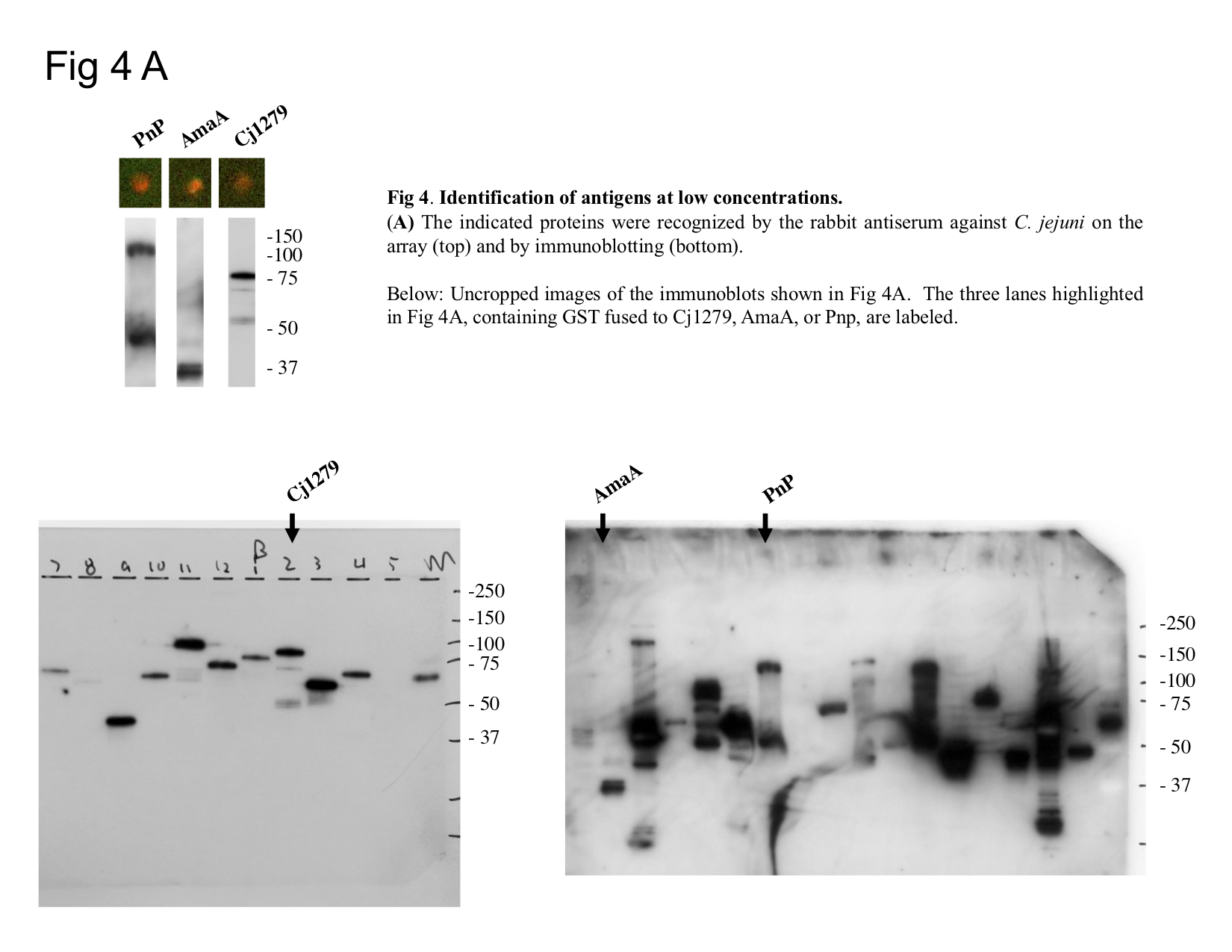

Supplement: S3 Fig — (TIFF) [file pone.0210351.s003.tiff]
